# Antibiotic Resistance and Genotypes of *Mycoplasma genitalium* during a Resistance-Guided Treatment Regime in a German University Hospital

**DOI:** 10.3390/antibiotics10080962

**Published:** 2021-08-10

**Authors:** Roger Dumke, Petra Spornraft-Ragaller

**Affiliations:** 1 Institut für Medizinische Mikrobiologie und Virologie, Universitätsklinikum, Technische Universität Dresden, Fetscherstrasse 74, 01307 Dresden, Germany; 2 Klinik und Poliklinik für Dermatologie, Universitätsklinikum, Technische Universität Dresden, Fetscherstrasse 74, 01307 Dresden, Germany; Petra.Spornraft-Ragaller@uniklinikum-dresden.de

**Keywords:** sexually transmitted infection, *Mycoplasma genitalium*, antibiotic resistance, genotyping, resistance-guided therapy

## Abstract

The treatment of infections from the sexually transmitted pathogen *Mycoplasma genitalium* is hampered by the rapidly increasing resistance to the recommended first- (macrolides) and second-line antibiotics (quinolones). Thus, resistance-guided therapy (RGT) is key for its successful eradication but the efficiency of this approach can be influenced by re-infections and treatment failures. The typing of strains is helpful to distinguish between ongoing colonization, re-infection or the development of resistance. In the present study, *mgpB* and MG_309 types as well as mutations associated with macrolide, quinolone and tetracycline resistance of strains in *M*. *genitalium*-positive samples accumulated in the years 2019 and 2020 at a university hospital were analyzed. Fifty-eight positive first and sixteen positive follow-up samples from patients (96.6% male, 84.5% men who have sex with men, 74.1% HIV-positive) were included. Twenty-three *mgpB* types (seven new types), nine MG_309 types and thirty-four *mgpB*/MG_309 types were identified. The prevalence of mutations associated with macrolide, quinolone and tetracycline resistance was 56.9%, 10.3% and 6.8%, respectively. Despite the fact that many asymptomatic patients were not treated and tests of cure were impossible in different cases, the preliminary rate of successful eradication (93.3%) in this study is promising for the continuation of the RGT strategy.

## 1. Introduction 

*Mycoplasma genitalium* is a Mollicutes species characterized by a strongly reduced genome (5.8 Mbp) resulting in limited metabolic capabilities and virulence factors. The most striking feature is the lack of a classic cell wall. Between humans, as the only known natural hosts, *M. genitalium* is sexually transmitted and occurs predominantly among people with high-risk sexual behaviors (e.g., men who have sex with men (MSM)) [1,2,3]. The microorganisms cause non-gonococcal urethritis (NGU) and are associated with cervicitis and pelvic inflammatory disease [4,5,6]. *M. genitalium* has been detected in 10 to 25% of patients with NGU resulting in persistent symptoms in up to 41% of men after treatment failure (reviewed in [4,6]). However, the evaluation of prevalence is difficult as many infected patients are asymptomatic [3,7,8,9]. Due to the intrinsic resistance to all betalactam antibiotics and the narrowed efficacy (22–45%) of doxycycline [10], options for the treatment of infections are limited. The European guideline for the treatment of *M. genitalium* infections [11] recommends azithromycin as first-line and moxifloxacin as second-line therapeutics. Unfortunately, the rate of strains with an acquired resistance has increased worldwide and has reached more than 50% (macrolides) and 10% (quinolones) especially in risk populations [12,13]. Recently, first strains showing mutations probably associated with a resistance to tetracycline were described [14,15], which, if spread in the future, could further affect the success of treatment concepts. Progress in the molecular characterization of the pathogen, investigations in cases of treatment failure and antimicrobial testing of the fastidiously growing bacteria in specialized laboratories in the last few years have resulted in the determination of resistance-associated mutations in 23S rRNA (macrolides) and *parC* (fluoroquinolones) of *M. genitalium* and the development of methods for the detection of these transitions [7,8]. Although cure rates >90% have been reached with a resistance-guided treatment (RGT) [16,17], the limited availability of approaches for resistance testing in ambulant settings hinders the establishment of an efficient regime to increase the nationwide eradication rates in practice. Furthermore, adherence problems, re-infections after unsafe sexual contacts, therapy failures and the development of resistance during therapy complicate the evaluation of the results of the recommended test-of-cure (ToC) samples. In this context, genotyping using a combination of determination of sequence differences in a highly variable region of the adhesin *MgpB* and of the number of repeats in gene MG_309 is a reliable tool to characterize strains in first and follow-up samples [18] and helps to differentiate between ongoing colonization (treatment failure, acquisition of resistance) or re-infection. To extend the current data on this common sexually transmitted pathogen, we determined resistance-associated mutations and genotypes in *M. genitalium* strains sampled during an RGT regime in a tertiary care hospital.

## 2. Results and Discussion

This study summarizes the initial results after the introduction of a program of an extended characterization of *M. genitalium* strains in a university hospital in Germany. The presence of a reference laboratory locally and the close cooperation with clinical facilities allowed the consistent testing of all *M. genitalium* strains for markers of resistance as well the registration of patient data and of corresponding antibiotic therapy. Table 1 summarizes the information about the patients included in the study and the results of the characterization of *M. genitalium* strains. Among the 58 patients (mean age: 41.4 years, range: 20–63 years), 96.6% (*n* = 56) were men and 74.1% (*n* = 43) were confirmed as HIV-positive. Forty-nine patients (84.5%, *n* = 49) identified themselves as MSM. As most of the patients were investigated during a regular screening for sexually transmitted infections in HIV-positive MSM, the majority of samples were rectal swabs (*n* = 37, 63.8%) followed by urethral swabs (*n* = 11, 19.0%), urine (*n* = 8, 13.8%) and vaginal swabs (*n* = 2, 3.4%), respectively. Symptoms (exclusively signs of urethritis) were reported in only eight cases (13.8%).

Mutations associated with macrolide resistance (MRAM) were found in 33 strains (56.9% of 58 patients). These were mainly A2059G (*Escherichia coli* numbering) transitions (75.8%, *n* = 25) whereas an A to G change at position 2058 was confirmed in samples from the remaining patients. In contrast to only one of nine non-MSM patients (11.1%), 65.3% (*n* = 32) of strains from MSM exhibited macrolide resistance (*p* = 0.004, OR 15.059, 95% CI 1.700–688.360). Interestingly, the strain in the urethral swab of a heterosexual man (#50) showed an unusual C2084T mutation of 23S rRNA. Due to a negative follow-up test after treatment with azithromycin, an association of this nucleotide change with macrolide resistance is unlikely. Mutations leading to quinolone resistance were confirmed in strains from six patients (10.3%) and resulted in an S83I (4×) or a D87Y change (2×) of the corresponding amino acid sequence of ParC (*M. genitalium* numbering). Four of these cases of quinolone resistance were combined with macrolide resistance resulting in a multi-drug resistance rate of 6.9%. Mutations in 16S rRNA of *M. genitalium* associated with tetracycline resistance (G966T + G967T, C1192G; *E. coli* numbering) were found in three patients (6.8%). Of note, a partial sequencing of 16S rRNA was successful in only 44 of 58 samples and was done retrospectively. Patients carrying tetracycline-resistant *M. genitalium* were HIV-positive MSM and these strains showed additional mutations associated with macrolide and quinolone resistance.

**Figure 1:**
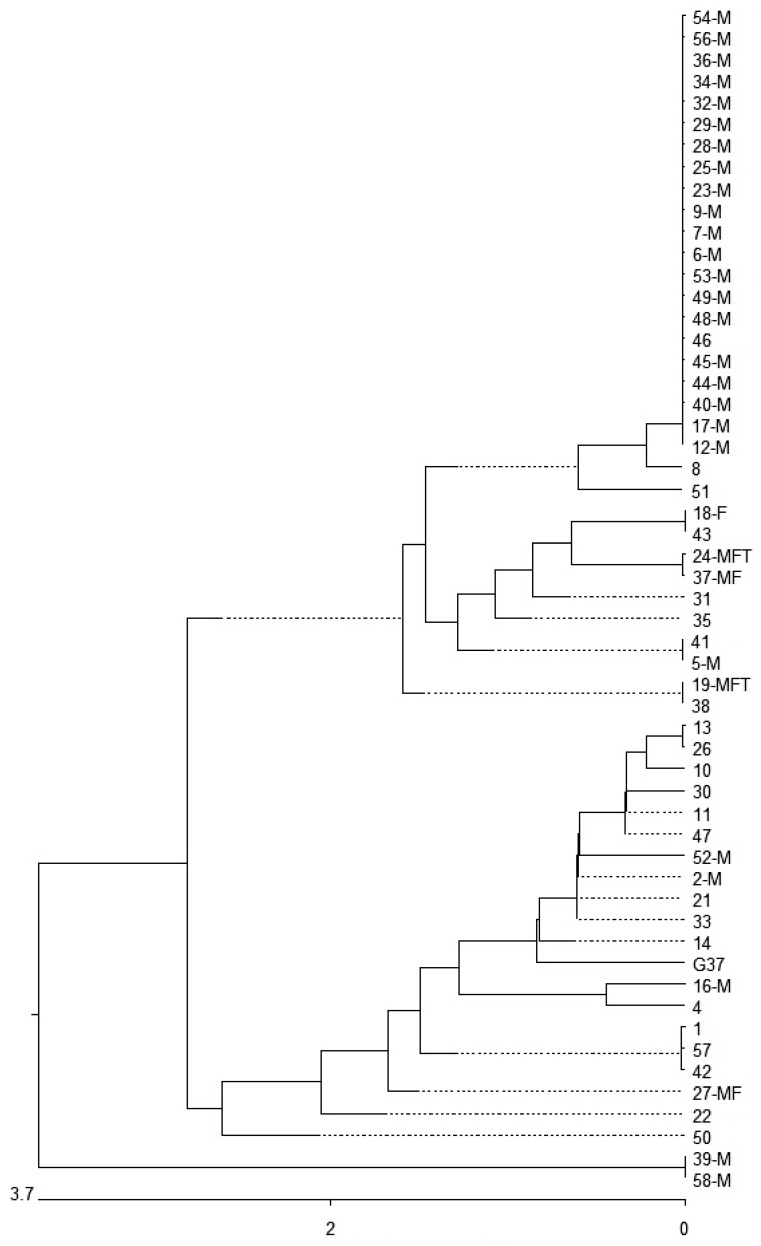
Similarity of partial *mgpB* sequences of type strain G37 and 54 *M. genitalium* clinical strains. M: strain with a macrolide resistance-associated mutation; F: strain with a fluoroquinolone resistance-associated mutation; T: strain with a tetracycline resistance-associated mutation. Bold and underlined: new types in comparison with the types described in Supplementary Table S1. Nucleotide substitution per 100 residues.

*MgpB* and MG_309 genotyping was successful in 93.1% (*n* = 54) and 91.4% (*n* = 53) of strains in the first samples. The combined *mgpB*-MG_309 type was found in 87.9% (*n* = 51) of cases. Overall, nine MG_309, 23 *mgpB* and 34 *mgpB*-MG_309 types were found (Table 1, Figure 1) resulting in discrimination indices [19] of 0.821, 0.844 and 0.953, respectively. Among the *mgpB* types, seven new types were identified (Table 2, Tables S1 and S2) and type 4 was most common (38.9%, *n* = 21). The phylogenetic tree of *mgpB* types shows that most of the strains (96.3%, *n* = 52) occurred in two clusters (Figure 1). Interestingly, the rate of macrolide resistance was 72.7% (24/33) in cluster 1 and only 21.0% (4/19) in cluster 2 (*p* = 0.0005, OR 10.0, 95% CI 2.256–50.374). Of the *mgpB* type 4 strains (belonging to cluster 1), 95% (20/21) were macrolide-resistant. All type 4-carrying patients were MSM and 71.4% (*n* = 15) of them were HIV-positive.

The recommended ToC was performed in 30 patients (51.7%; including the above-mentioned patient #50). All of the 16 ToC-tested patients carrying strains without resistance-associated mutations showed a *M. genitalium*-negative PCR (13 were treated with azithromycin and three with doxycycline because of accompanying sexually transmitted infections). Among the 14 patients infected with macrolide-resistant strains, 9 were successfully treated with moxifloxacin and 3 with doxycycline. Two patients remained positive after treatment with doxycycline (#15 and 29, positive follow-up samples #2f and 6f). Interestingly, in a man carrying a strain with a combined resistance to macrolides, quinolones and tetracyclines (patient #27), the ToC was negative after therapy with doxycycline. Overall, the ToC was negative in 28 of the 30 treated patients (93.3%).

Sixteen follow-up samples from eleven patients were *M. genitalium*-positive (Table 3) in the investigation period. The type of specimen corresponded with the first sampling. The interval between the first and subsequent positive sample ranged between 8 and 85 weeks. According to their asymptomatic status, only four of these patients were treated between the first and follow-up sample. In three cases (patients #2, 5 and 7), a negative test result between both positive samples was demonstrated, suggesting re-infection in the time before the second positive test. This hypothesis was partly supported by the results of genotyping. In comparison with the first sampling, the strain in specimen #1f showed the same genotype but differed in the occurrence of the mutation in 23S rRNA; in sample #7f, differences in the presence of MRAMs and in the *mgpB*-MG_309 type were found whereas the strain in #10f was identical in all available resistance and typing markers. As *mgpB*-MG_309 typing has been characterized as highly discriminating and stable in different studies [18,20,21], low-level colonization or sampling problems might be the reasons for the negative result of the intermediate rectal swab in the latter patient. Importantly, patients #2 and 5 received moxifloxacin in the period before the second positive test. Due to co-infections, doxycycline was prescribed before follow-up sampling in cases #2f and 6f. In sample #6f, the available results of genotyping were identical between the samples. In the sample from one patient without an antibiotic therapy between sampling (#3f), differences in at least one genotyping marker suggest a time-dependent colonization with genetically different strains whereas the available resistance and typing pattern of the strains of both samples in the remaining three patients without treatment (samples #14f, 15f and 16f) were identical. Multiple *M. genitalium*-positive follow-up samples from three patients (#15, 19 and 26) were analyzed (interval between the samples: up to 53 weeks). In the samples from patient #19, the available results of the occurrence/non-occurrence of resistance-associated mutations and *mgpB*/MG_309 typing were identical, indicating a long-term colonization (52 weeks) with the same multi-resistant *M. genitalium* genotype. Due to sequencing failures, specimens from patient #15 were only partially comparable whereas samples from patient #26 showed genotype differences between the strains in the first and second positive samples but the genotyping pattern remained identical in the further positive samples (up to 38 weeks).

The increasing rates of resistance of *M. genitalium* to the recommended first- and second-line antibiotics, including the occurrence of multi-drug-resistant strains, are a cause for concern for public health. To control infections, not only new antimicrobials but also extended surveillance programs, a wider availability of resistance testing and optimized treatment strategies are necessary [6,7,8]. This is especially the case for particular populations. With the high rate of HIV-positive people and of MSM, the investigated patients in this study belonged to high-risk groups for the acquisition of *M. genitalium* infections and for colonization with resistant strains [22,23,24,25]. This fact hampers the comparison of resistance rates and genotypes between these groups and those of the few non-MSM and HIV-negative *M. genitalium* carriers included in the study. The overall rates of macrolide and quinolone resistance of 56.9% and 10.3% were in the range of reports from other countries [12,13]. Comparable studies in Germany are rare. Recent data from an MSM-dominated population in Berlin, Germany, demonstrated a similar rate of quinolone resistance (10.4%) but a higher rate of macrolide-resistant strains (79.9%), indicating local differences in the antibiotic susceptibility of *M. genitalium* strains [25]. Practitioners and public health authorities must anticipate the variability of the resistance pattern of circulating strains depending not only on whether patients belong to risk groups but also on regional characteristics. These findings further emphasize the value of resistance tests prior to antibiotic treatment.

As doxycycline is not recommended among first-, second- and third-line antibiotics to treat *M. genitalium* infections [11], investigations to detect mutations of tetracycline-binding sites in 16S rRNA probably associated with resistance are scarce. Furthermore, the importance of particular transitions for the resistance of strains has been derived from other species and is not confirmed by susceptibility tests with *M. genitalium* or by using subinhibitory concentrations of doxycycline to induce corresponding mutations in the 16S rRNA of *M. genitalium*. In two recent reports from France [14,15], mutations associated with a resistance to tetracyclines were found in 6 of 106 strains (5.7%) and 2 of 16 strains (12.5%). Despite its unusual use for the treatment of *M. genitalium* infections, doxycycline is part of an RGT scheme developed in Australia to reduce the bacterial load until the result of resistance testing of the strain is available [16] or is recommended as a component of a non-quinolone option for treatment [26]. If the rate of tetracycline-resistant strains is increasing, the use of tetracycline as an alternative treatment as part of RGT and for post-exposure prophylaxis may be further limited.

In addition to their importance for the interpretation of *M. genitalium*-positive follow-up tests, genotyping results provide interesting aspects regarding the circulation of distinct types in human populations. In the present study, more than one-third of strains could be assigned to *mgpB* type 4 in cluster 1 (all of them found in MSM) and this type is disproportionately associated with macrolide resistance. Despite the low rate of non-MSM with a confirmed *mgpB* type among the patients (*n* = 8, 13.8%), one of two women who have sex with men (WSM) and the men who have sex with women (MSW) belonged to cluster 2 (exception: woman #35), supporting the hypothesis of a largely independent circulation of genotypes in both groups. This was also shown in a recent study [27]. After the investigation of strains sampled between the years 2014 and 2016 in Dresden (31.6%) and between 2017 and 2018 in Berlin (38.6%), type 4 was also the most common *mgpB* type [21,28]. Furthermore, the results of reports from France and Spain demonstrated rates of type 4 strains of 23.8% and 27.8%, respectively [20,27]. All these studies included a high percentage of MSM. In contrast, the frequency of genotype 4 strains remains relatively low (<7%) if populations with presumably low rates of MSM are analyzed, as shown in Australia, South Africa and Spain [29,30,31]. The findings suggest an increased circulation of *mgpB* type 4 among MSM, at least in Europe, which is linked to a high rate of macrolide resistance. However, the rate of macrolide-resistant strains in the present study (56.9%) was lower in comparison with that found in Berlin [25]. Further nationwide studies are necessary to substantiate this preliminary result. Nevertheless, it can be assumed that the occurrence of *M. genitalium* strains among carriers with a particular preferred sexual behavior is polyclonal and appears to be different to that of other populations. In combination with a high rate of the prescription of macrolides among (HIV-positive) MSM, the genotypes in this specific group tend to demonstrate a higher rate of resistance to azithromycin. At this stage, it remains unclear if the predominance of *mgpB* type 4 among MSM is the result of easier transmission during anal intercourse [20] or of the limited connectivity between the sexual networks of MSM and WSM/MSW [27].

This study has several limitations. Despite the more common occurrence in symptomatic men, rectal colonization with *M. genitalium* was described as asymptomatic in many patients [9]. Strains in all *M. genitalium*-positive samples within the study period were tested at our hospital for resistance-associated mutations; a decision on the antibiotic therapy of patients depended on the presence of symptoms and the possibility of partner treatment to avoid a development of resistance, antibiotic side effects and unnecessary treatment costs [8,32]. Furthermore, the exact rate of the spontaneous clearing of rectal *M. genitalium* colonization in asymptomatic patients is not known. Thus, the number of positive but untreated patients is relatively high. Secondly, the treatment of patients was carried out exclusively in outpatient departments of the hospital. Unfortunately, this fact is often associated with a relatively high number of patients missing for a ToC (e.g., further treatment in other offices) or their late re-appearance causing difficulties in evaluating the overall eradication success of RGT. The investigation of resistance markers especially *mgpB*/MG_309 typing demonstrates on the one hand the possibility of the long-term colonization of patients (e.g., patient #19) with strains showing identical resistance and typing markers but also of patients becoming infected with genetically different strains during the investigation period. Depending on the sexual behavior, both patterns of colonization have been described in previous studies [20,21,33,34].

In conclusion, the preliminary data of the present study confirm the high rate of antibiotic resistance among *M. genitalium* strains in a local group of patients with an increased risk of becoming infected with this pathogen. Furthermore, the results of typing demonstrate the polyclonal structure of the investigated strains with a dominance of *mgpB* type 4, which is associated with a frequent occurrence in MSM and macrolide resistance. Compared with non-MSM patients, being MSM causes a 15-fold increase in the odds of harboring macrolide-resistant strains. The rates of tetracycline- and quinolone-resistant strains were relatively low but need further monitoring. Despite the detection of re-infections, which might influence the evaluation of RGT strategies, the results underline the importance of subsequent and early susceptibility testing to control the transmission of *M. genitalium*.

## 3. Materials and Methods

In this study, we collected all *M. genitalium*-positive samples between January 2019 and December 2020 in the University Hospital of Dresden, Germany. The gender and HIV status (if available) of patients, reported presence of symptoms, location of sampling and results of the follow-up tests were recorded. DNA was extracted using an automated system (EZ1; Qiagen, Hilden, Germany) and *M. genitalium* was detected by real-time PCR as recommended by the manufacturer (TIB MolBiol, Berlin, Germany). The DNA of positive samples was frozen at −80 °C until a further analysis. Overall, strains in 58 first and 16 positive follow-up samples were characterized. Mutations in 23S rRNA and the *parC* gene of *M. genitalium* associated with a resistance to macrolides and quinolones as well as *mgpB*-MG_309 genotypes were determined by PCR/Sanger sequencing as described previously [21,25]. Nucleotide changes in a part of the 16S rRNA of *M. genitalium* were determined by a semi-nested PCR as reported [35] and using a new forward primer for the first amplification (MG16-1f, 5′-GCA ATG CCG CGT GAA CGA TGA AGG-3′). All obtained sequences were compared with the corresponding regions in the reference genome of strain G37 (GenBank accession no. NC_000908.2). Due to the small numbers, a two-sided Fisher’s exact test was performed for the comparison of phylogenetic *M. genitalium* clusters 1 and 2 as well as for the comparison of MSM and non-MSM patients regarding the frequency of macrolide resistance. The confidence interval of the OR was calculated from a logit model. For calculation, Stata version 15.1 (StataCorp LLC, College Station, TX, USA) was used.

## Figures and Tables

**Table 1:** Characteristics of patients and *M. genitalium* strains.

Patient No.	Gender ^1^	MSM/MSW/WSM ^2^	HIV Status	Symptoms	Sample ^3^	MRAM ^4^	FRAM ^5^	TRAM ^6^	Genotype ^7^
1	m	MSM	pos. ^8^		US	no ^9^	no	no	**231**–13
2	m	MSW	n.t. ^10^		US	A2058G	no	no	2–14
3	m	MSM	neg. ^11^		U	A2058G	no	no	n.d. ^12^–11
4	m	MSM	pos.		RS	no	no	no	134–10
5	m	MSM	pos.		RS	A2059G	no	n.d.	111–10
6	m	MSM	pos.		RS	A2059G	no	n.d.	4–10
7	m	MSM	pos.		RS	A2059G	no	n.d.	4–10
8	m	MSM	pos.		U	no	no	no	62–10
9	m	MSM	pos.		RS	A2059G	no	no	4–10
10	m	MSM	pos.		RS	no	no	no	**232**–11
11	m	MSM	pos.		R	no	no	n.d.	5–12
12	m	MSM	pos.		U	A2059G	no	n.d.	4–n.d.
13	m	MSW	neg.		US	no	no	no	133–9
14	m	MSW	n.t.		US	no	no	no	125–10
15	m	MSM	pos.		RS	A2059G	no	n.d.	n.d.–n.d.
16	m	MSM	pos.		U	A2059G	no	no	6–12
17	m	MSM	pos.		RS	A2059G	no	no	4–11
18	m	MSM	pos.		U	no	S83I	n.d.	**233**–n.d.
19	m	MSM	pos.		RS	A2058G	D87Y	C1192G	113–11
20	m	MSM	pos.		U	A2059G	no	no	n.d.–11
21	f	WSM	n.t.		VS	no	no	no	2–14
22	m	MSM	pos.		U	no	no	no	**234**–14
23	m	MSM	pos.	Urethritis	US	A2059G	no	no	4–10
24	m	MSM	pos.		RS	A2058G	S83I	G966T and G967T	3–13
25	m	MSM	neg.		RS	A2059G	no	no	4–11
26	m	MSM	pos.		RS	no	no	n.d.	8–12
27	m	MSM	pos.		RS	A2058G	D87Y	C1192G	12–13
28	m	MSM	pos.		RS	A2059G	no	n.d.	4–11
29	m	MSM	pos.	Urethritis	RS	A2059G	no	n.d.	4–12
30	m	MSW	n.t.		US	no	no	no	5–11
31	m	MSM	pos.		RS	no	no	no	**235**–10
32	m	MSM	pos.		RS	A2058G	no	no	4–10
33	m	MSW	n.t.	Urethritis	US	no	no	no	2–12
34	m	MSM	neg.		RS	A2059G	no	no	4–10
35	f	WSM	pos.		VS	no	no	no	**236**–11
36	m	MSM	neg.	Urethritis	RS	A2059G	no	no	4–10
37	m	MSM	pos.		RS	A2058G	S83I	n.d.	3–n.d.
38	m	MSM	pos.		RS	no	no	no	113–14
39	m	MSM	pos.		RS	A2059G	no	no	52–18
40	m	MSM	neg.		RS	A2059G	no	no	4–9
41	m	MSM	pos.		RS	no	no	n.d.	111–11
42	m	MSM	pos.		RS	no	no	no	**231**–16
43	m	MSM	pos.		RS	no	no	n.d.	**233**–12
44	m	MSM	pos.		RS	A2059G	no	no	4–10
45	m	MSM	pos.		RS	A2059G	no	no	4–11
46	m	MSM	pos.	Urethritis	US	no	no	no	4–12
47	m	MSM	pos.		RS	no	no	no	5–9
48	m	MSM	pos.		RS	A2059G	no	no	4–9
49	m	MSM	pos.		RS	A2059G	no	no	4–10
50	m	MSW	neg.		US	no	no	no	7–11
51	m	MSM	pos.		RS	no	no	no	**237**–9
52	m	MSM	pos.		RS	A2059G	no	no	24–12
53	m	MSM	neg.	Urethritis	US	A2059G	no	no	4–11
54	m	MSM	neg.		RS	A2059G	no	no	4–11
55	m	MSW	n.t.	Urethritis	US	no	S83I	n.d.	n.d.–n.d.
56	m	MSM	pos.	Urethritis	U	A2059G	no	no	4–11
57	m	MSM	pos.		RS	no	no	no	**231**–14
58	m	MSM	pos.		RS	A2058G	no	no	52–19

^1^—f: female, m: male; ^2^—women/men who have sex with men/women; ^3^—RS: rectal swab, U: urine, US: urethral swab, VS: vaginal swab; ^4^—macrolide resistance-associated mutation (nucleotide exchange in 23S rRNA of *M. genitalium*, *Escherichia coli* numbering); ^5^—fluoroquinolone resistance-associated mutation (amino acid exchange in ParC of *M. genitalium*); ^6^—tetracycline resistance-associated mutation (nucleotide exchange in 16S rRNA of *M. genitalium*, *E. coli* numbering); ^7^—*mgpB* type number of repeats in MG_309, bold and underlined: new *mgpB* types; ^8^—positive; ^9^—no mutations; ^10^—not known/not tested; ^11^—negative; ^12^—not determined (sequencing failure, limited sample volume).

**Table 2:** Distribution of *mgpB* types of 54 *M. genitalium* clinical strains.

MgpB Type	Number of Strains (%)
4	21 (38.9)
2	3 (5.6)
5	3 (5.6)
**231**	3 (5.6)
3	2 (3.7)
52	2 (3.7)
111	2 (3.7)
113	2 (3.7)
**233**	2 (3.7)
6	1 (1.8)
7	1 (1.8)
8	1 (1.8)
12	1 (1.8)
24	1 (1.8)
62	1 (1.8)
125	1 (1.8)
133	1 (1.8)
134	1 (1.8)
**232**	1 (1.8)
**234**	1 (1.8)
**235**	1 (1.8)
**236**	1 (1.8)
**237**	1 (1.8)

bold and underlined: new *mgpB* types.

**Table 3:** Characteristics of *M. genitalium* strains in follow-up samples.

*M. genitalium*-Positive Follow-Up Samples (Belonging to Patient No. in Table 1, Previous Follow-Up Sample No.)	Weeks after Previous Test	Previous Antibiotic Treatment	Negative Test between First and Follow-Up Sample	MRAM	FRAM	TRAM	Genotype
1f (2)	18	MOX ^1^	yes	no	no	no	2–14
2f (15)	8	DOX ^2^	no	A2059G	no	n.d.	4–11
3f (13)	12		no	no	no	no	3–8
4f (19)	26		no	A2058G	D87Y	C1192G	113–11
5f (26)	15		no	A2059G	no	no	4–10
6f (29)	13	DOX	no	A2059G	no	no	4–12
7f (5)	52	MOX	yes	no	no	n.d.	4–11
8f (15, 3f)	34		no	A2059G	no	no	4–n.d.
9f (26, 6f)	12		no	A2059G	no	no	4–10
10f (7)	59		yes	A2059G	no	no	4–10
11f (19, 5f)	26		no	A2058G	D87Y	C1192G	113–11
12f (26, 6f, 10f)	17		no	A2059G	no	no	4–10
13f (26, 6f, 10f, 13f)	9		no	A2059G	no	no	4–10
14f (48)	27		no	A2059G	no	no	4–9
15f (24)	59		no	A2058G	S83I	G966T and G967T	3–13
16f (12)	85		no	A2059G	no	no	4–10

^1^—moxifloxacin; ^2^—doxycycline.

## Data Availability

The data presented in this study are available on reasonable request from the corresponding author. The data are not publicly available due to data protection reasons.

## Supplementary Materials

The following are available online at https://www.mdpi.com/article/10.3390/antibiotics10080962/s1, Table S1 (designation of *M. genitalium* strains based on *mgpB* typing) and Table S2 (sequences of new *mgpB* types) [36].
