# Supplementary material for: Antibiotic Resistance and Genotypes of Mycoplasma genitalium during a Resistance-Guided Treatment Regime in a German University Hospital
Source: Antibiotics (Basel). 2021 Aug 10;10(8):962. doi: 10.3390/antibiotics10080962 (PMC8389038; doi:10.3390/antibiotics10080962)
Supplement: Supplementary file 1 [file antibiotics-10-00962-s001.zip › antibiotics-1313951-supplementary.pdf]

antibiotics

MDPI

*Communication*

# Antibiotic Resistance and Genotypes of *Mycoplasma Genitalium* during a Resistance-Guided Treatment Regime in a German University Hospital

**Roger Dumke [1,*] and Petra Spornraft-Ragaller [2]**

[1] Institut für Medizinische Mikrobiologie und Virologie, Universitätsklinikum, Technische Universität Dresden, Fetscherstrasse 74, 01307 Dresden, Germany
[2] Klinik und Poliklinik für Dermatologie, Universitätsklinikum, Technische Universität Dresden, Fetscherstrasse 74, 01307 Dresden, Germany; Petra.Spornraft-Ragaller@uniklinikum-dresden.de
* Correspondence: roger.dumke@tu-dresden.de

**Table S1.** Designation of *M. genitalium* strains based on *mgpB* typing.

| Proposed Type Description | References |
|---|---|
| 1–160 | [1] |
| 161–183 | [2] |
| 184–189 | [3] |
| 190–208 | [4] |
| 209–212 | [5] |
| 213–230 | [6] |
| 231–237 | Present study |

**Table S2.** Sequences of new *mgpB* types.

Type 231 (patients #1, 42, 57)

ACTTGAAACAATAACAACTTCTCTTCACTAAAGATTACTGGAGAGAACCCAGGATCATTT-

GGATTAGTAAGAAGCCAAAATGACAACTTAAACATCGCAAGTGTTACAAAGAATGTTAGTGATGATAATCTCA

AGTATCTCAATACTGTTGAGAAATACCTTGATGGTCAGCAAAACTTT-

GCAATCAGAAGGTATGATAACAACGGTAGAGCTTTATATGATATTAACTTAGCAAAAATGGAAAACCCCTCAA

CGG

Type 232 (patient #10)

ACTTGAAACAATAACAACTTCTCTTCACTAAAGATTACTGGAGAGAACCCAGGATCATTT-
GGATTAGTAAGAAGCCAAAATGACAACTTAAATATTTCAAATGTTATTACAAAGAATAATGTTAGTGGTGATA
ATCTCAAGTATCTCAATGATGTTGAGAAATACCTTGATGGTCAGCAAAACTTT-
GCAATCAGAAGGTATGATAACAACGGTAGAGCTTTATATGATATTAACTTAGCAAAAATGGAAAACCCCTCAA
CGG

Type 233 (patients #18, 43)

ACTTGAAACAATAACAACTTCTCTTCACTGAAGATTACTGGAGAGAACCCAGGATCATTT-
GGACTAGTAAGAAGCCAAAATGAGAACTTAAACATCGCAAGTGTTACAAAGGATGGTAATGGTGATAATCTC
AAGTATCTTAATGCTGTTGAGAAGTACCTTGATGGTCAGCAAAACTTT-
GCAATCAGAAGGTATGATAACAACGGTAGAGCTTTATATGATATTAACTTAGCAAAAATGGAAAACCCCTCAA
CGG

Type 234 (patient #22)

ACTTGAAACAATAACAACTTCTCTTCACTAAAGATTACTGGAGAGAACCCAGGATCATTT-
GGATTAGTAAGAAGCCAAAATGACAACTTAAACATCGCAAGTGTTACAAAGAATGTTAGTGATGATAATCTCA
AGTATCTTAATTCTGTTGAGAAATACCTTGATGGTCAGCAAAACTTT-
GCAATCAGAAGGTATGATAACAACGGTAGAGCTTTATATGATATTAACTTAGCAAAAATGGAAAACCCCTCAA
CGG

Type 235 (patient #31)

ACTTGAAACAATAACAACTTCTCTTCACTGAAGATTACTGGAGAGAACCCAGGATCATTT-
GGACTAGTAAGAAGCCAAAATGAGAACTTAAACATCGCAAGTGTTACAAAGAATAGTAGTGATGATAATCTC
AAGTATCTTAATGCTGTTGAGAAGTACCTTGATGGTCAGCAAAACTTT-
GCAATCAGAAGGTATGATAACAACGGTAGAGCTTTATATGATATTAACTTAGCAAAAATGGAAAACCCCTCAA
CGG

Type 236 (patient #35)

ACTTGAAACAATAACAACTTCTCTTCACTAAAGATTACTGGAGAGAACCCAGGATCATTT-
GGACTAGTAAGAAGCCAAAATGAGAACTTAAACATCGCAAGTGTTACAAAGAATGGTAGTGATGATAATCTC

AAGTATCTTAATGCTGTTGAGAAGTACCTTGATGGTCAGCAAAACTTT-

GCAATCAGAAGGTATGATAACAACGGTAGAGCTTTATATGATATTAACTTAGCAAAAATGGAAAACCCCTCAA

CGG

Type 237 (patient #51)

ACTTGAAACAATAACAACTTCTCTTCACTAAAGATTACTGGAGAAAACCCAGGATCATTT-

GGACTAGTAAGAAGCCAAAATGAGAACTTAAACATCGCAAGTGTTACAAAGAATGATAGTGATGATAATCTC

AAGTATCTTAATGCTGTTGAGAAATACCTTGATGGTCAGCAAAACTTT-

GCAATCAGAAGGTATGATAACAACGGTAGAGCTTTATATGATATTAACTTAGCAAAAATGGAAAACCCCTCAA

CGG

## References


1. Dumke R, Rust M, Glaunsinger T. *MgpB* types among *Mycoplasma genitalium* strains from men who have sex with men in Berlin, Germany, 2016-2018. *Pathogens* **2019**, *20*, 9:12.
2. Guiraud J, Lounnas M, Boissière A, Le Roy C, Elguero E, Banuls AL, Bébéar C, Godreuil S, Pereyre S. Lower mgpB diversity in macrolide-resistant *Mycoplasma genitalium* infecting men visiting two sexually transmitted infection clinics in Montpellier, France. *J. Antimicrob. Chemother*. **2021**, *76*, 43-47.
3. Laumen JGE, van Alphen LB, Maduna LD, Hoffman CM, Klausner JD, Medina-Marino A, Kock MM, Peters RPH. Molecular epidemiological analysis of *Mycoplasma genitalium* shows low prevalence of azithromycin resistance and a well-established epidemic in South Africa. *Sex. Transm. Infect*. **2021**, *97*, 152-156.
4. Fernández-Huerta M, Serra-Pladevall J, Esperalba J, Moreno-Mingorance A, Fernández-Naval C, Barberá MJ, Aparicio D, Pich OQ, Pumarola T, Jensen JS, Espasa M. Single-locus-sequence-based typing of the *mgpB* gene reveals transmission dynamics in *Mycoplasma genitalium*. *J. Clin. Microbiol*. **2020**, *58*, e01886-19.
5. Plummer EL, Murray GL, Bodiyabadu K, Su J, Garland SM, Bradshaw CS, Read TRH, Tabrizi SN, Danielewski JA. A custom amplicon sequencing approach to detect resistance associated mutations and sequence types in *Mycoplasma genitalium*. *J. Microbiol. Methods* **2020**, *179*, 106089.
6. Sweeney EL, Tickner J, Bletchly C, Nimmo GR, Whiley DM. Genotyping of *Mycoplasma genitalium* suggests *de novo* acquisition of antimicrobial resistance in Queensland, Australia. *J. Clin. Microbiol*. **2020**, *58*, e00641-20.
